# Immunoreactivity and neutralization study of Chinese *Bungarus multicinctus* antivenin and lab-prepared anti-bungarotoxin antisera towards purified bungarotoxins and snake venoms

**DOI:** 10.1371/journal.pntd.0008873

**Published:** 2020-11-30

**Authors:** Bo Lin, Jia-Rui Zhang, Hui-Juan Lu, Lin Zhao, Jing Chen, Hong-Fei Zhang, Xue-Song Wei, Liang-Yu Zhang, Xiao-Bing Wu, Wen-Hui Lee

**Affiliations:** 1 College of Life Sciences, Anhui Normal University, Wuhu, Anhui, China; 2 Key Laboratory of Animal Models and Human Disease Mechanisms of The Chinese Academy of Sciences/Key Laboratory of Bioactive Peptides of Yunnan Province, Kunming Institute of Zoology, the Chinese Academy of Sciences, Kunming, Yunnan, China; 3 Nanshan School, Guangzhou Medical University, Guangzhou, Guandong, China; 4 School of Life and Pharmaceutical Sciences, Hainan University, Haikou, Hainan, China; Instituto de Biomedicina de Valencia, SPAIN

## Abstract

*Bungarus multicinctus* is the most venomous snake distributed in China and neighboring countries of Myanmar, Laos, north Vietnam and Thailand. The high mortality rate of *B*. *multicinctus* envenomation is attributed to the lethal components of α-, β-, γ- and κ- bungarotoxins contained in the venom. Although anti-*B*. *multicinctus* sera were produced in Shanghai, Taiwan and Vietnam, the most widely clinic used product was term as *B*. *multicinctus* antivenin and manufactured by Shanghai Serum Bio-technology Co. Ltd. In the present investigation, high purity α-, β- and γ-bungarotoxins were separately isolated from *B*. *multicinctus* crude venom. Rabbit anti- α-, β- and γ-bungarotoxin antisera were prepared by common methods, respectively. LD_50_ values of α-, β- and γ-bungarotoxins were systematically determined via three administration pathways (intraperitoneal, intramuscular and intravenous injections) in Kunming mice. LD_50_ values of β-bungarotoxin were closely related with injection routines but those of both α- and γ-bungarotoxins were not dependent on the injection routines. Commercial *B*. *multicinctus* antivenin showed strong immunoreaction with high molecular weight fractions of the *B*. *multicinctus* but weakly recognized low molecular weight fractions like α- and γ-bungarotoxins. Although *B*. *multicinctus* antivenin showed immunoreaction with high molecular weight fractions of *Bungarus fasciatus*, *Naja atra*, *Ophiophagus hannah* venoms but the antivenin only demonstrated animal protection efficacy against *O*. *hannah* venom. These results indicated that the high molecular weight fractions of the *O*. *hannah* played an important role in venom lethality but those of *B*. *fasciatus* and *N*. *atra* did not have such a role.

## Author summary

Snakebite envenoming is an important public health problem around the world. *Bungarus multicinctus* envenomation is regarded as most dangerous animal bitten disease in China and neighboring countries. At present, the most widely clinic used *B*. *multicinctus* antisera was term as *B*. *multicinctus* antivenin and manufactured by Shanghai Serum Bio-technology Co. Ltd. By using high purity α-, β- and γ-bungarotoxins isolated from *B*. *multicinctus* crude venom and prepared corresponding rabbit anti- α-, β- and γ-bungarotoxin antisera, we evaluated the efficacy of *B*. *multicinctus* antivenin against the crude venom, isolated bungarotoxins, as well as other 8 medical important Chinese venomous snake venoms. *B*. *multicinctus* antivenin showed strong immunoreaction with high molecular weight fractions of the *B*. *multicinctus* venom but weakly recognized low molecular weight fractions like α- and γ-bungarotoxins. Moreover, *B*. *multicinctus* antivenin can neutralize *Ophiophagus hannah* venom but not *B*. *fasciatus* venom was demonstrated.

## Introduction

The study of toxins and venomous animals has a long history, it affects human life and health [1]. Snakebite envenoming is an important public health problem around the world and was classified as category A neglected tropical disease in 2017 [2]. Recently, a 5 × 5 km resolution contemporary range maps covering global medical important 278 snake species were provided and the vulnerability to snakebite envenoming in subnational scale was given precisely. Combined with antivenom availability, accessibility to urban centers as well as healthcare access and quality index, authors identify the most vulnerable to snakebite morbidity and mortality populations. Unfortunately, China ranks the first in country level count of vulnerable people living within the range of one or more medically important venomous snake species and as many as 33,499,658 Chinese people affected nowadays [2]. It was reported that up to 2.7 million snakebites occur worldwide per year which were responsible for 81,000–138,000 deaths annually as well as 400,000 amputations and other permanent disabilities [2–4]. At present, national level snakebite and venomous snake envenomation epidemiology of mainland China is still largely unknown. A comparative comprehensive snakebite epidemiological survey of Guangxi Zhuang Autonomous Region, one of the provincial level snakebite hotspots in southern China, was carried out co-operatively by Guangxi Medical University and Japan Snake Institute. The investigation results indicated that 37,600 snakebites occurred and 1019 deaths each year in Guangxi around 1990s. Further analysis of the epidemiological data revealed that there were 993 snakebite cases happened in Guangxi in 1990. Farmers 715 cases (72%), students 100 cases (10.1%) and workers 84 cases (8.5%) were the most vulnerable victims. Only 62 cases received antivenom serum treatment at that time and totally 27 victims died from snake-bite out of the 993 cases were reported. Notably, 4 cases out of 62 antivenom serum treatment cases were eventually died because of delayed treatment, in which 3 cases were bitten by *Bungarus multicinctus* and 1 case by *Bungarus fasciatus* [5]. However, the number of snakebites might be underestimated because snakebites often occurred in remote, poor rural or mountainous areas that many cases of snakebite unreported.

There are more than 60 species of venomous snakes in China, of which 10 most harmful species are regarded as follows: *B*. *multicinctus*, *B*. *fasciatus*, *Naja atra*, *Ophiophagus hannah*, *Deinagkistrodon acutus*, *Gloydius brevicaudus*, *Trimeresurus stejnegeri*, *Daboia russelii siamensis*, *Protobothrops mucrosquamatus* and *Laticauda colubrina* [6]. Among the Chinese top ten venomous snakes, *B*. *multicinctus* represents the most venomous snake in mainland China. It was reported that *B*. *multicinctus* bites accounted for 8.12% of all snakebites in China but the lethality of *B*. *multicinctus* bite ranked the first [7]. *B*. *multicinctus* widely distributed in south China and neighboring countries of Myanmar, Laos, north Vietnam and Thailand globally. The high mortality rate of *B*. *multicinctus* envenomation is attributed to presynaptic neurotoxins and postsynaptic neurotoxins contained in the venoms, which can block neurotransmitter at motor nerve terminals and eventually leads to respiratory failure and death [8].

The high lethal effects of *B*. *multicinctus* are attributed to neurotoxins existed in the venom. *B*. *multicinctus* venom contains four different lethal neurotoxic components classified as α-bungarotoxin (α-BGT), β-bungarotoxin (β-BGT), γ-bungarotoxin (γ-BGT) and κ-bungarotoxin (κ-BGT). β-BGT is composed by a phospholipase A_2_ subunit and a kunitz-type protease inhibitor subunit linked by an S-S band. β-BGT acts as presynaptic toxin with phospholipase A_2_ subunit that is similar to most presynaptic neurotoxin while the kunitz-type protease inhibitor subunit plays a key role to guide the toxin to its target [9–11]. α-BGT, γ-BGT and κ-BGT act on postsynaptic membrane. α-BGT mainly interacts with α7 acetylcholine receptor and GABA receptor [12–13]. γ-BGT and κ-BGT act on M2 and α3β2 acetylcholine receptors, respectively. All of the three postsynaptic toxins can directly block the nerve transmission between neuromuscular junction [14–17].

In present investigation, we focus on the neutralization capacity evaluation of the commercial *B*. *multicinctus* antivenin produced in mainland China. Systematical LD_50_ values of the purified bungarotoxins via three administration routines in Kunming mice were also determined.

## Materials and methods

### Ethics statement

All experiments on animals meet the requirements of National Institutes of Health guide for the care and use of Laboratory animals (NIH Publications No. 8023) and has been reviewed and approved by Animal Care and Use Committee of Kunming Institute of Zoology, Chinese Academy of Sciences (Approval ID: SMKX-2017023).

Kunming mice (male, 20 ± 2 g) and rabbit (2.5 kg) were provided by Hunan SJA Laboratory Animal Co., Ltd. The animals were fed in animal house of Kunming Institute of Zoology, Chinese Academy of Sciences with a dark/photo period of 12 hours.

### Venoms and antivenin

Pooled *B*. *multicinctus* venom was purchased from a snake farm in Zhejiang Province, China. Snake venoms of *B*. *fasciatus*, *N*. *atra*, *O*. *hannah*, *D*. *acutus*, *T*. *stejnegeri*, *D*. *russelii siamensis*, *G*. *brevicaudus* and *P*. *mucrosquamatus* were stocks of our lab at Kunming Institute of Zoology, the Chinese Academy of Sciences. All the used venoms are collected from corresponding adult snakes which are born and grow in snake farms. *B*. *multicinctus* antivenin was purchased from Shanghai Serum Bio-technology Company (Batch No. S10820179, Expire date: 16/03, 2020). Presently, only four kinds of monovalent antivenins are available in mainland China as follows: *B*. *multicinctus* (10000 U/vial), *Naja atra* (1000 IU/vial), *G*. *brevicaudus* (6000 U/vial) and *D*. *acutus* (2000 U/vial) antivenins. All of them are equine immune globulin F(abʹ)_2_ fragments digested by gastric enzymes and provided in liquid solutions (10 ml/vial). All of the 4 kinds of monovalent antivenins are produced by Shanghai Serum Bio-technology Co. Ltd, the sole anti-snake venom sera producer in mainland China (http://www.serum-china.com.cn/enprodetail/187.html).

### Purification of α-, β- and γ-BGTs

Lyophilized *B*. *multicinctus* venom (250 mg) was dissolved in 2 ml 0.05 M PBS (pH 7.0) and centrifuged at 10,000 g for 20 min at 4°C. Then the supernatant was loaded onto a Superdex G75 gel filtration column (100×2.6 cm, XK 26, GE Health, USA) which was equilibrated with the same buffer and eluted at a flow rate of 1 ml/min on an Akta Purifier system (GE Healthcare, Uppsala, Sweden). For β-BGT purification, the peak IV of gel filtration was collected and dialyzed against large volume of 0.05 M sodium acetate-acetic acid buffer, pH 5.0. Then, the dialyzed sample was loaded onto a source 15S cationic ion-exchange chromatography column (16×2 cm, GE Health, USA) pre-equilibrated with the same buffer. Elution was carried out at a flow rate of 2 ml/min by a NaCl gradient. The target fraction was collected and further purified by the source 15S cationic ion-exchange chromatography column with 0.02 M phosphate buffer, pH 7.3. Finally, the target fraction collected from second ion-exchange step was freeze-dried and dissolved in Milli-Q water before being loaded onto a reverse C_18_ HPLC column (300×4.6 mm, Hypersil BDS, Elites, Daliang, China). The C_18_ column was pre-equilibrated with 0.1% (v/v) trifluoroacetic acid (TFA) and eluted with an acetonitrile (containing 0.1% TFA) gradient. For α-BGT and γ-BGT purification, the peak V of gel filtration was collected and treated the same as the first ion exchange step of β-BGT purification. High purity α-BGT and γ-BGT were isolated from peak II and peak IV of the ion exchange steps by a single HPLC step on the reverse C_18_ HPLC column, respectively.

### Construction and expression of maltose binding protein fusion γ-BGT

The amount of γ-BGT contained in the *B*. *multicinctus* venom is too low to get enough toxin to immunize rabbits. Thus, a maltose binding protein (MBP) fusion γ-BGT was constructed and expressed in prokaryotic expression system to prepare γ-BGT antiserum. Briefly, the mature peptide sequence of γ-BGT was retrieved from UniProt (UniProt: Q9YGJ0). The synthetic gene coding for γ-BGT was optimized and inserted into the pMAL-p2X (using BamH I and Sac I) vectors [New England Biolabs (Beijing) Ltd., Beijing, China] by Shanghai Generay Biotech Co., Ltd (Shanghai, China). Finally, the MBP-γ-BGT expression plasmid was subcloned into *Escherichia coli* (TB1). The TB1 culture was shaken at 180 rpm, 37°C, IPTG was added at a final concentration of 0.5 mM when the OD_600_ of the culture medium reach 0.6. The cultures were grown for 24 h in shaking flasks at 180 rpm, 16°C. The recombinant MBP-γ-BGT protein was successfully expressed in the periplasm of TB1. The periplasmic fractions were extracted according to the protocols provided by the manufacturer and the fusion protein was purified by amylose affinity resin [New England Biolabs (Beijing) Ltd., Beijing, China].

### Protein quantification

Protein quantification was determined by Pierce BCA assay kit (Thermo Scientific, Rockford, USA) using bovine serum albumin as standard, according to manufacturer’s instructions.

### Mass spectrometry

The mass spectrometry analysis was performed according to the reported methods [18–19]. Briefly, the molecular weights of purified bungarotoxins were determined by matrix-assisted laser desorption ionization time-of-flight mass spectrometry (AUTOFLEX III MALDI-TOF, Bruker Corporation, Germany). For *de novo* mass sequencing of internal peptides, purified neurotoxins were separately isolated by SDS-PAGE under non-reducing conditions. Then, the target bands were cut and digested by trypsin at 37°C. The detailed sample handling was the same as our recently reported methods [18]. Operation was carried out on positive ion mode using α-Cyano-4-hydroxycinnamic acid as matrix. The MS/MS spectrums were analyzed by Biotools software provided by the manufacturer and combined with manual annotation for MS/MS spectra interpretation.

### SDS-PAGE and western blotting

SDS-PAGE was performed according to the method of Laemmli [20]. The gel was stained with Coomassie blue R-250 and de-stained with 10% acetic acid in 40:50 (v/v) water/ethanol solution. Different snake venoms were first run on a 15% SDS-PAGE gel under reducing or non-reducing conditions and then transferred onto a PVDF membrane (0.45 μm, Millipore). Then, each PVDF membrane was subsequently blocked with 5% nonfat milk in TBST (20 mM Tris-HCl, pH 7.4, contained 0.5% Tween-20) for 2 h at room temperature. The PVDF membrane was completely washed in TBST and incubated with antivenin or our prepared antiserum (1:5000 dilution) overnight at 4°C. The PVDF membrane was washed three times in TBST to remove the primary antibody and then incubated with 1:5000 dilution secondary antibody at room temperature for 2 h. Finally, the membrane was visualized by using SuperSignal West Pico Chemiluminescent Substrate Kit (34080, Thermo Scientific, IL, USA). Normal horse and rabbit IgG used for commercial *B*. *multicinctus* antivenin and prepared antiserum controls were products from Proteintech.

### Median lethal dose (LD_50_) determination

The LD_50_ value was determined according to the method of Meier & Theakston [21]. Briefly, groups of 6 mice were used per dose for each sample and injected in a final volume of 200 μl through intravenous (*i*.*v*), intraperitoneal (*i*.*p*) and intramuscular (*i*.*m*) routines. The LD_50_ values were further accurately determined using different sample concentrations around the approximate LD_50_ for different routines within 24 hours after administration, respectively.

### Rabbit specific antiserum preparation

The purified β-BGT, α-BGT and recombinant MBP-γ-BGT protein were separately used to immune rabbits according to common methods. Briefly, β-BGT and α-BGT were diluted to a final concentration of 2 mg/ml with PBS containing 0.4% (w/v) formaldehyde for detoxification and kept in dark for 7 days at 37°C until completely detoxified. Then, each detoxified toxin (1.5 ml) and recombinant MBP-γ-BGT protein (2 mg/ml, 1.5 ml) was separately mixed with an equal volume of Freund’s complete adjuvant and used for the first subcutaneous immunization on rabbits. After that, each sample was mixed with an equal volume of Freund’s incomplete adjuvant for subsequent subcutaneous immunization every 7 days, four immunizations were performed afterward. Finally, polyclonal antisera were purified from rabbit blood by a Protein A Sepharose column [New England Biolabs (Beijing) Ltd., Beijing, China], respectively [22]. Freund’s complete adjuvant and incomplete adjuvant used were products from Sigma-Aldrich (MO, USA).

### Immunoreactivity study of commercial antivenin and prepared antisera

The immunoreactivity of antisera was defined as half of the maximum dilution factor at which the immunological binding can be observed [23]. Briefly, 100 μl of *B*. *multicinctus* venom, purified β-BGT, α-BGT and γ-BGT at a constant final concentration of 10 μg/ml were separately coated to a 96-well ELISA plate (Corning, USA) overnight at 4°C. After blocking by 3**%**BSA (B2064, Simga-Aldrich), different dilutions of specific antiserum (1:1,000~500,000) were added to the plate (100 μl/well) and incubated 2 h at 37°C. Then, 1:5000 diluted goat against horse the secondary antibody (for commercial antivenin) or goat against rabbit the secondary antiserum (for our prepared α-BGT, β-BGT and γ-BGT) were separately added to each well and incubated at 37°C for 1 h. All secondary antibodies were labeled with horseradish peroxidase (HRP) and bought from Proteintech (SA00001, USA). Finally, the absorbance at 450 nm was obtained on an Infinite Tecan M200 Pro micro-plate Reader (Austria) after adding HRP detecting substrates provided by Beyotime Biotechnology (P0209, China) according to manufacturer’s instructions.

### Determination of 50% effective doses (ED_50_)

According to WHO recommendation, the neutralizing capability of the antivenom in our experiments was expressed as 50% effective doses (ED_50_) that the dose of antivenom required to protect 50% of mice when injected with 3LD_50_ of venom [24]. Briefly, mice were intraperitoneal injected with a mixture (200 μl) of different doses of rabbit antisera containing 3LD_50_ of *B*. *multicinctus* venom (n = 6/group), the mixture was incubated at 37°C for 30 min prior to inject. The mice were observed up to 48 h after injection. The ED_50_ values were calculated according to the Spearman-Karber equation as follows [25–27]:

$$\log ED_{50}=\log x_{100}-\frac{\log FD}{n}\left(\sum t-\frac{n}{2}\right)$$

Definition of term: log X100 = log dose giving 100% survival and having 100% survival for all higher doses. log FD = the log dilution factor. n = mice used at each dose level. t = mice alive at each dose level. Σ = the sum of mice surviving at every dose level. The weight of mice used in antivenin protection test is calculated as 20 g. ED_50_ values of antivenoms were expressed as mg of antivenoms per kg body weight of mouse to neutralize the challenge dose of venom.

## Results

### Purification and characterization of α-, β- and γ-BGTs

**Fig 1:**
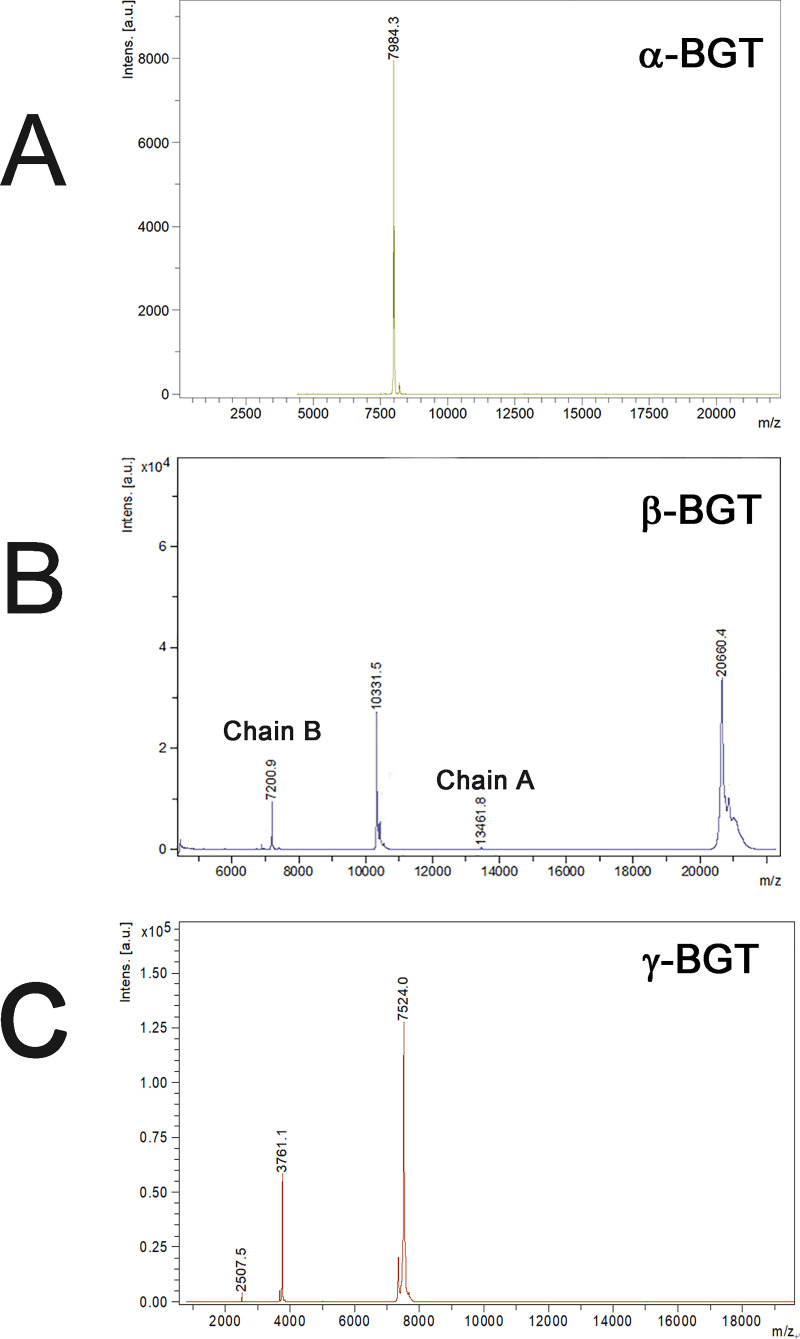
Molecular weight determination of purified bungarotoxins by MALDI/TOF mass spectrometer. α-BGT (A). β-BGT (B). γ-BGT (C).

By a combination of gel filtration, ion exchange and HPLC procedures, high purity α-, β- and γ-BGTs were isolated from *B*. *multicinctus* venom. The amounts of the isolated α-, β- and γ- BGTs from 1 g of *B*. *multicinctus* venom were estimated to be about 30 mg, 10 mg and 0.3 mg, respectively (S1 Fig). β-BGTs were mainly existed in peak IV of gel filtration step as the elution peaks (I-VIII) of the first ion exchange of gel filtration peak IV (S1B Fig) all gave β-BGT signal in SDS-PAGE (S3 Fig). Finally, we isolated a high purity β-BGT by additional 2 purification steps (S1C and S1D Fig). Both α-BGT and γ-BGT existed in peak V of gel filtration step and could be easily purified by an ion exchange step followed by a HPLC step, respectively (S1E–S1G Fig). The determined amino acid sequences of purified bungarotoxins by *de novo* MS/MS sequencing match well with corresponding toxins found in UniProt database (S2A–S2C Fig). The determined masses of purified β-BGT, α-BGT and γ-BGT were 20660.4 Da, 7984.3 Da and 7524.0 Da, respectively (Fig 1). Meanwhile, 13461.8 Da and 7200.9 Da corresponding to A-chain and B-chain of purified β-BGT were also determined (Fig 1B). Combing molecular weight and *de nove* internal peptide sequence determinations, the purified α-BGT and γ-BGT are identical to UniProt database of P60615 and Q9YGJ0, respectively. However, only the kunitz-type protease inhibitor subunit identical to UniProt database of P00989 was found for purified β-BGT, the phospholipase A_2_ subunit could not match that of known identified β-BGTs but the *de nove* sequences of FGNSEYIEGHKNIDTAR and TIICYGAAGTCGR conserved in the known phospholipase A_2_ subunit of β-BGTs. Blast search results suggested that the beta-bungarotoxin A chain of our purified β-BGT best match that of UniProt P00617.

The recombinant MBP-γ-BGT protein was expressed in TB1 and successively purified from periplasmic fractions by an affinity column (S4 Fig). Approximately 50 mg MBP-γ-BGT protein can be obtained from 1 liter of the culture medium. The purified recombinant MBP-γ-BGT fusion protein had no lethal activity when injected *i*.*v* at high dose of 1 mg/mouse.

### Median lethal dose (LD_50_) determination

The LD_50_ values of α-BGT, β-BGT and γ-BGT were determined to be 0.2 μg/g, 0.004 μg/g and 0.091 μg/g via *i*.*p* injections, 0.24 μg/g, 0.015 μg/g and 0.088 μg/g via *i*.*m* injections, 0.17 μg/g, 0.007 μg/g and 0.074 μg/g via *i*.*v* injections, respectively (Table 1). Table 1 also provided currently available LD_50_ data on bungarotoxins, a slight difference of the data might be caused by the test animal used [28–38].

**Table 1:** Summary of the available LD_50_ values of bungarotoxins via different administration routines.

	LD_50_ (μg/g)				Types of mice	Reference
	*i*.*p*	*i*.*m*	*i*.*v*	subcutaneous		
*B*. *multicinctus* venom	**0.09**	-	-	-	**Kunming mice**	**Present work**
	-	-	-	0.16	Mice (15–20 g)	Chang CC (1963)
	0.08	-	0.071	-	White mice (20–25 g)	Kocholaty WF (1971)
	-	-	0.014	-	ICR mice (20–25 g)	Ratanabanangkoon K (2016)
α-BGT(A31)	**0.2**	**0.24**	**0.17**	-	**Kunming mice**	**Present work**
α-BGT	0.23	-	-	-	Outbreeding mice(18–20 g)	Kuch U (2003)
α-BGT	0.14	-	-	-	NIH strain(20–22 g)	Wu SH (1983)
α-BGT	-	-	-	0.3	Mice (15–20 g)	Chang CC (1963)
α-BGT	-	-	-	0.14	Swiss mice	Eterovic VA (1975)
α-BGT	0.11	-	-	-	Swiss mice	
α-BGT	0.25	-	-	-	Female ICR mice (20–30 g)	Crosland RD (1989)
β-BGT	**0.004**	**0.015**	**0.007**	-	**Kunming mice**	**Present work**
β-BGT	-	-	-	0.089	Mice (15–20 g)	Chang CC (1963)
β1-BGT	0.019	-	-	-	mice	Kondo K(1982)
β2-BGT	0.028	-	-	-		
β3-BGT	0.066	-	-	-		
β4-BGT	0.072	-	-	-		
β5-BGT	0.013	-	-	-		
SPI	0.123	-	-	-	Albino mice (20–25 g)	Chu CC (1994)
SPII	0.043	-	-	-		
SPIII	0.012	-	-	-		
β-BGT	-	-	0.05	-	Swiss mice (20–25 g)	Rosenberg P (1989)
β-BGT	0.0097	-	-	-	Female ICR mice (20–30 g)	Crosland RD (1989)
γ-BGT	**0.091**	**0.088**	**0.074**	-	**Kunming mice**	**Present work**
γ-BGT	-	-	0.15	-	Swiss mice	Aird SD (1999)
γ-BGT	-	-	-	0.12	Mice (15–20 g)	Chang CC (1963)
κ-BGT	-	-	-	-		

Abbreviations: α-BGT, α-bungarotoxin; β-BGT, β-bungarotoxin; γ-BGT, γ-bungarotoxin; κ-BGT, κ-bungarotoxin; SP I, SP II and SP III are different subtypes of β-bungarotoxin; “-”, no data available.

### Immunoreactivity of bungarotoxin-specific antisera

**Fig 2:**
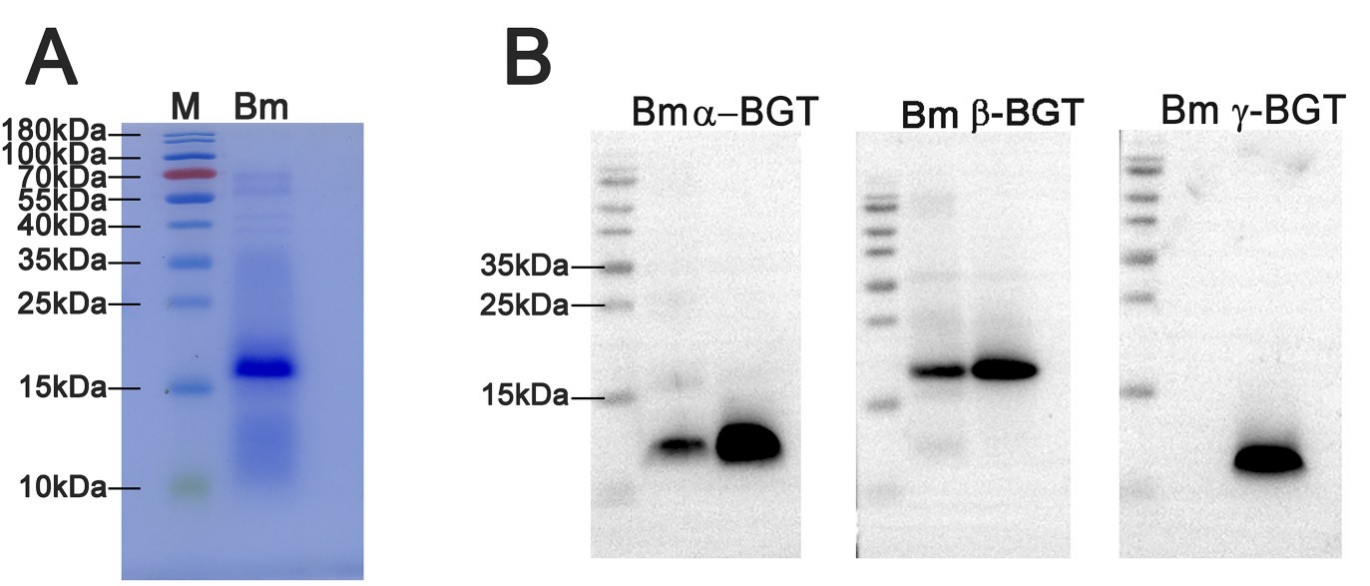
Specificity of three prepared antisera. SDS-PAGE of *B*. *multicinctus* venom under non-reducing conditions, 25 μg/sample (A). Western-blot profile of prepared anti-α-BGT, anti-β-BGT and anti-γ-BGT antisera against crude venom and corresponding bungarotoxins (B). For Western-blot, 2 μg of crude venom or purified neurotoxins were added in each lane. Bm: Crude venom of *B*. *multicinctus*.

Both prepared anti-β-BGT and anti-α-BGT antisera could well recognize crude venom and corresponding purified β-BGT or α-BGT, respectively. However, anti-γ-BGT antiserum could only recognize purified γ-BGT but not crude venom because the γ-BGT contained in the crude venom was too low to be detected (Fig 2B).

**Fig 3:**
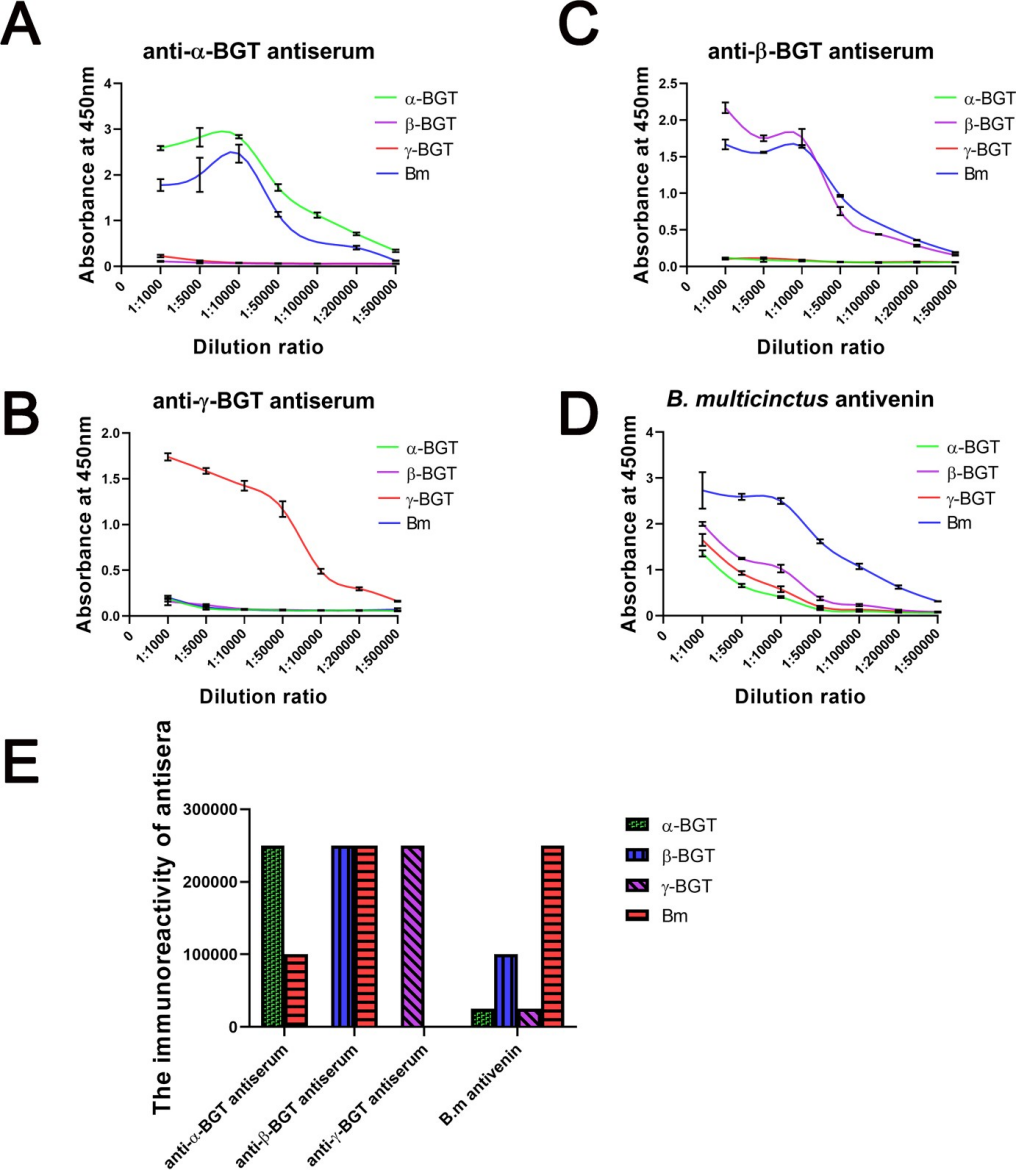
The immunoreactivity of antisera comparison by indirect ELISA. Prepared anti-α-BGT antiserum against different antigens (A). Prepared anti-γ-BGT antiserum against different antigens (B). Prepared anti-β-BGT antiserum against different antigens (C). Commercial *B*. *multicinctus* antivenin against different antigens (D). The immunoreactivity determination of prepared antisera and the *B*. *multicinctus* antivenin (E). Antigens of 1μg/well were coated on a 96-well plate, results were expressed as mean ± SD.

To further characterize the specificity of our prepared antisera, the absorbance at 450 nm of different antiserum against different antigens were determined by indirect ELISA experiments. A fixed concentration of 2 mg/ml was used for both antisera and *B*. *multicinctus* antivenin. The results revealed that the specificities of all the prepared rabbit antisera were quite well since no cross-reaction with other purified bungarotoxins was detected for each prepared antiserum (Fig 3A–3C). Again, present used commercial *B*. *multicinctus* antivenin showed better neutralizing capacity against crude venom, but obvious weak immunoreactivity against γ-BGT and α-BGT were found (Fig 3D). The immunoreactivity determination demonstrated that the prepared anti-α-BGT antiserum well recognized purified α-BGT but showed weak immunoreactivity against crude venom. Meanwhile, the prepared anti-β-BGT antiserum had the same immunoreactivity when immunobinding with *B*. *multicinctus* venom and purified β-BGT. Notably, the immunoreactivity of the commercial *B*. *multicinctus* antivenin against *B*. *multicinctus* venom was 2.5 times higher than that of immunobinding with purified β-BGT. *B*. *multicinctus* antivenin showed very weak immunoreactivity against purified α-BGT and γ-BGT (Fig 3E).

### Immunoreactivity of the commercial *B*. *multicinctus* antivenin and prepared antisera

**Fig 4:**
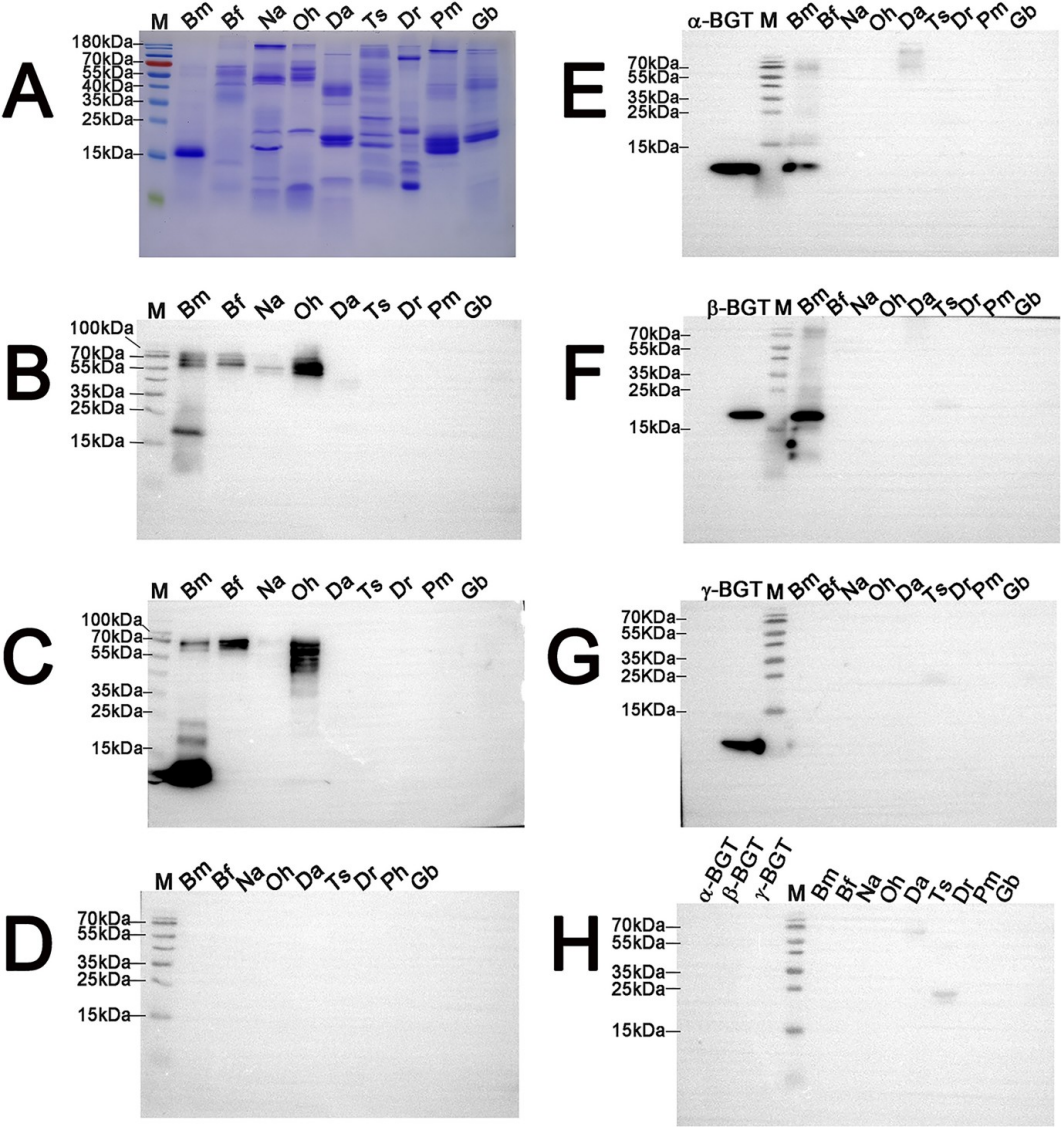
Immunoreactivity of the commercial *B*. *multicinctus* antivenin and the prepared antiserum against top Chinese dangerous terrestrial snake venoms. SDS-PAGE of nine used snake venoms under non-reducing conditions, 25 μg/sample (A). Western blot revealed by *B*. *multicinctus* antivenin under non-reducing conditions (B). Western blot revealed by *B*. *multicinctus* antivenin under reducing conditions (C). Western blot revealed by normal horse IgG under non-reducing conditions (D). Western blot revealed by prepared anti-α-BGT antiserum under non-reducing conditions (E). Western blot revealed by prepared anti-β-BGT antiserum under non-reducing conditions (F). Western blot revealed by prepared anti-γ-BGT antiserum under non-reducing conditions (G). Western blot revealed by normal rabbit IgG under non-reducing conditions (H). Bm: *B*. *multicinctus*; Bf: *B*. *fasciatus*; Na: *N*. *atra*; Oh: *O*. *hannah*; Da: *D*. *acutus*; Ts: *T*. *stejnegeri*; Dr: *Daboia russelii siamensis*; Pm: *P*. *mucrosquamatus*; Gb: *G*. *brevicaudus*. 10 μg/sample was loaded for Western blot experiments.

To investigate whether commercial *B*. *multicinctus* antivenin has immunological binding activities with other venomous terrestrial snake venoms, eight other dangerous Chinese snake venoms were used. SDS-PAGE stained with Coomassie blue under non-reducing conditions showed the natural profiles of proteins/peptides contained in the nine used snake venoms (Fig 4A). The immunoreactivity between venoms and commercial *B*. *multicinctus* antivenin had obvious difference under reducing or non-reducing conditions. High immunoreactivity existed in *B*. *multicinctus* high MW (molecule weights) bands (~55–100 kDa) and ~20 kDa β-BGT fractions; similar high MW bands (~ 55–100 kDa) also found in *B*. *fasciatus* but not 20 kDa fractions; a weak band of ~ 55 kDa exited in *N*. *atra*; strong immunoreactivity in high MW bands (~ 55–70 kDa) was found in *O*. *hannah* under non-reducing conditions, respectively (Fig 4B). However, high immunoreactivity existed in *B*. *multicinctus* low MW (13 kDa) corresponding to A-chain of β-BGT and high MW bands (~ 55–70 kDa); high MW bands (~55–70 kDa) still found in *B*. *fasciatus*; no signal in *N*. *atra*; strong immunoreactivity in high MW bands (~ 45–70 kDa) in *O*. *hannah* were observed under reducing conditions, respectively (Fig 4C). No immunoreactivity was detected for the *B*. *multicinctus* antivenin against other used snake venoms under reducing or non-reducing conditions (Fig 4B and 4C). Since anti-serum recognized antigens better under non-reducing conditions, the cross-neutralization evaluation of prepared antiserum was carried out under non-reducing conditions. The results indicated that the specificities of the prepared antiserum were quite well and no cross-reactions with other snake venoms were found (Fig 4E–4G).

### ED_50_ values of the commercial used *B*. *multicinctus* antivenin and prepared antisera

According to the determined LD_50_ values of the *B*. *multicinctus* venom and purified bungarotoxins, the protection capability (ED_50_) of each antiserum was separately calculated by injection with 3LD_50_ of different toxins via *i*.*p* injections (Table 2).

**Table 2:** ED_50_ determination of *B*. *multicinctus* antivenin and the prepared bungarotoxin antiserum against *B*. *multicinctus* venom and purified bungarotoxins.

Antiserum	Toxins	LD_50_ (mg/kg) *i*.*p*^a^	Antiserum Dose (μg/mouse)	Number of mice (n = 6)		ED_50_^a^ (mg/kg)
				Died	Lived	
*B*. *multicinctus* antivenin	*B*. *multicinctus* venom	0.09	62.5	6	0	**17.68**
			125	6	0	
			250	6	0	
			500	0	6	
	α-BGT	0.2	1000	6	0	**178.18**
			2000	6	0	
			4000	2	4	
			8000	0	6	
	β-BGT	0.004	2.5	6	0	**0.5**
			5	5	1	
			10	4	2	
			20	0	6	
β-BGT antiserum	*B*. *multicinctus* venom	0.09	62.5	6	0	**12**
			125	6	0	
			250	3	3	
			500	0	6	
	β-BGT	0.004	2.5	6	0	**0.314**
			5	4	2	
			10	1	5	
			20	0	6	
α-BGT antiserum	α-BGT	0.2	62.5	6	0	**11.14**
			125	4	2	
			250	4	2	
			500	0	6	
	*B*. *multicinctus* venom	0.09	4000	6	0	**>200**
α-BGT antiserum plus β-BGT antiserum (1:1)	*B*. *multicinctus* venom	0.09	62.5	6	0	**17.68**
			125	6	0	
			250	6	0	
			500	0	6	

a ED_50_ values of antivenoms were expressed as mg of antivenoms per kg body weight of mouse to neutralize the challenge dose of venom.

Moreover, since commercial *B*. *multicinctus* antivenin also showed immunoreactivity against *B*. *fasciatus*, *N*. *atra* and *O*. *hannah* venoms, the ED_50_ values of *B*. *multicinctus* antivenin against these three Elapidae venoms were also determined (Table 3).

**Table 3:** The protective effects of antiserum against different snake venoms.

antiserum	venoms	LD_50_ (mg/kg) *i*.*p*^a^	Antivenin Dose (μg/mouse)	Number of mice		ED_50_^b^ (mg/kg)
				Died	Lived	
*B*. *multicinctus* antivenin	*Bungarus fasciatus*	1.5	1000	6	0	**>800**
			2000	6	0	
			8000	6	0	
			16000	5	1	
	*Naja atra*	0.5	1000	6	0	**>800**
			2000	6	0	
			8000	6	0	
			16000	5	1	
	*Ophiophagus hannah*	0.44	1000	6	0	**400**
			2000	6	0	
			8000	3	3	
			16000	0	6	

a LD_50_ values of the crude venoms were determined by present work in Kunming mice.

b ED_50_ values of antivenoms were expressed as mg of antivenoms per kg body weight of mouse to neutralize the challenge dose of venom.

## Discussion

The first edition of “China Expert Consensus on the management of snake-bites” launched in 2018. The morbidity and mortality of snake-bite are still high in mainland China nowadays. Shortages of antivenom and overdependence on folk medicine to treat snake-bites are common in China, especially in rural areas and remote mountainous areas [39]. Snake species of *B*. *multicinctus*, *B*. *fasciatus*, *N. atra*, *O*. *hanna*h, *D*. *acutus*, *G*. *brevicaudus*, *T*. *stejnegeri*, *D*. *russelii siamensis*, *P*. *mucrosquamatus* as well as sea snake of *L*. *colubrina* are listed as the top 10 venomous snakes in mainland China. Among them, *B*. *multicinctus* is regarded as the most virulent and deadly species [12]. According to recent snakebite epidemiological investigation in China, farmers still have been the largest proportion of snakebite victims (65%-68%) while the percentage of snake-dealers is about 2.4%-6.94% [40–43]. An act, issued by Chinese government on thoroughly banning the illegal trading of wildlife and eliminating the consumption of wild animals, has initiated since Feb. 24, 2020 in China. Consequently, the morbidity of bitten by venomous snakes might enhance in the near future since China is still an agricultural country. Thus, it is helpful to evaluate current available *B*. *multicinctus* antivenin against this most dangerous snake species in mainland China.

It is well known that lethal components responsible for the high morbidity and mortality of *B*. *multicinctus* envenomation are attributed to β-, α-, γ- and κ- BGTs contained in the venom, the latter three bungarotoxins all belong to three finger toxin (TFT) family [29,44]. Except γ-BGT, the rest three kinds of bungarotoxins all have various isoforms. High purity α-, β- and γ-BGTs isolated by currently reported methods greatly facilitate systemic LD_50_ assays and specific antiserum preparations. Unfortunately, κ-BGT was not purified in present work because the used crude venom might not contain this component, as mass determination of Superdex 75 gel filtration peak IV and peak V gave no κ-BGT signal (data not known). Interestingly, quantitative proteomic analysis as well as their components of *B*. *multicinctus* crude venoms were independently investigated by different research groups. β-BGT and TFT quantities contained in the crude venoms were reported to be 58.3%-32.6% (Guangxi, China) and 45%-28% (Vietnam), respectively [45–46]. However, γ-BGT was not detected in their used crude venoms. Taken together, β- and α- BGTs were constant contained in the *B*. *multicinctus* crude venoms, but γ- and κ-BGTs had the greatest variations in the crude venoms.

Previously, LD_50_ values of *B*. *multicinctus* crude venoms and isolated bungarotoxins were determined by different labs and most of them were carried out via single injection routines. For crude venom LD_50_ determination, a significant difference was observed by different groups via *i*.*v* injections (0.071 *vs* 0.014 μg/g). This difference might be caused by crude venom or experimental animals used. For isolated α-BGTs, the determined LD_50_ values varied weakly among different labs via different routines. However, the determined LD_50_ values of β-BGTs varied greatly. Notably, different β-BGTs showed obvious differences were found by different labs even using same tested animals (Table 1). Thus, using the same animals to evaluate toxins via different routine might be helpful to preciously explain the experimental results. Present work reported the systemic investigated the LD_50_ values of purified bungarotoxins via *i*.*p*, *i*.*m* and *i*.*v* injection routines in Kunming mice. Notably, administration routines did have effects on the lethal activity of bungarotoxins were first demonstrated. β-BGT had the highest lethal activity via *i*.*p* injection followed by *i*.*v* and *i*.*m* injections, respectively. Four times lethal activity difference existed in β-BGT via different injection routines. On the contrary, both α-BGT and γ-BGT showed the highest lethal activity via *i*.*v* injection. The lethal activity of both α-BGT and γ-BGT did not related to injection routines since the determined results were almost at the same levels for each of them. However, approximate 2–3 times high lethal activities of γ-BGT over α-BGT were found for different injection routines (Table 1). The calculated ED_50_ values of commercial *B*. *multicinctus* antivenin against *B*. *multicinctus* venom and α-BGT were 17.68 mg/kg and 178.18 mg/kg, respectively. ED_50_ value of prepared β-BGT antiserum against *B*. *multicinctus* venom was 12 mg/kg. ED_50_ value of prepared α-BGT antiserum against purified α-BGT was 11.14 mg/kg. Interestingly, ED_50_ value of equal quantity of prepared α-BGT and β-BGT antisera against *B*. *multicinctus* venom was identical to that of the clinic used *B*. *multicinctus* antivenin. Definitely, β-BGT is the highest lethal component of the crude venom, its ED_50_ values show 50 and 22.75 times via *i*.*p*, 150 and 5.86 times via *i*.*m*, 24 and 10 times stronger over α-BGT and γ-BGT, respectively (Table 2). Meanwhile, the ED_50_ value of *B*. *multicinctus* antivenin against *O*. *hannah* venom was determined to be 400 mg/kg, but maximal *B*. *multicinctus* antivenin used (original solution without dilution) could not protect *B*. *fasciatus* and *N*. *atra* envenomations and ED_50_ >800 mg/kg were given for these 2 venoms (Table 3).

At present, commercial anti-*B*. *multicinctus* serum was produced in mainland China (Shanghai), Taiwan and Vietnam [47]. However, WHO recommended *B*. *multicinctus* antivenom were produced by Shanghai and Taiwan (https://apps.who.int/bloodproducts/snakeantivenoms/database/). The *B*. *multicinctus* antivenom was termed as *B*. *multicinctus* antivenin by Shanghai Serum Bio-technology Company and is the only commercially available medicine in China (except Taiwan). *B*. *multicinctus* antivenin was monovalent F(abʹ)_2_ fragments prepared by traditional methods on horses and used in present works. To better evaluate the efficiency of *B*. *multicinctus* antivenin, anti-α-BGT antiserum and anti-β-BGT antiserum were prepared by using purified components as antigens in rabbits, anti-γ-BGT antiserum was prepared by using recombinant MBP-γ-BGT.

The best way to treat snakebite is to use specific antivenom in time. However, lacking of antivenoms or no specific antivenom available was common in clinic. This problem is very obvious in China since only 4 kinds of commercial antivenin available but approximately 60 venomous snake species existed. Thus, commercial antivenin is recommended to be used in clinic to treat snakebite patients of closely related snake species [48–49]. For example, “China Expert Consensus on the management of snake-bites” suggested using *B*. *multicinctus* antivenin to treat *B*. *fasciatus* victims, *B*. *multicinctus* plus *N*. *atra* antivenin to treat king cobra patients [39]. Thus, it’s necessary and helpful to evaluate the cross immunoreactivity of the available antivenom to prevent misuse of antivenoms in clinic. Present investigation evaluated immunoreactivity of commercial *B*. *multicinctus* antivenin and prepared antisera with eight other dangerous Chinese terrestrial snake venoms. The immunoreactivity between venoms and clinic *B*. *multicinctus* antivenin had obvious difference under reducing or non-reducing conditions. Surprisingly, current used *B*. *multicinctus* antivenin showed very strong immunoreactivity with high MW of *B*. *multicinctus* venom under non-reducing condition but very weak immunoreactivity with these fractions under reducing conditions. These results consistent with the conclusion that *B*. *multicinctus* antivenin well recognize crude venom but poorly recognize α-BGT, β-BGT and γ-BGT as depicted in Fig 1D. Previously, Gao et al evaluated the immunoreactivity between four venoms and commercial antivenoms produced by Shanghai Serum Bio-technology Company [50]. Comparing their western-blotting results of *B*. *multicinctus* antivenin against *B*. *multicinctus* crude venom with present results, a big difference existed in the immunoreactivity of *B*. *multicinctus* antivenin recognize *B*. *multicinctus* high MW fractions. The *B*. *multicinctus* antivenin used by Gao et al reacted strongly with β-BGTs but not high MW fractions under non-reducing conditions, which was contrary with our present results. The most possible reason might be caused by the *B*. *multicinctus* antivenin used by Gao et al was further purified by a HiTrap Protein G column or caused by the used crude venoms in producing of different batches of the antivenins. Although *B*. *multicinctus* antivenin could recognize high MW of *B*. *fasciatus* venom both under non-reducing and reducing conditions, these fractions were not the lethal components of the venom and ED_50_ >800 mg/kg demonstrating this conclusion. *B*. *multicinctus* antivenin showed very weak reaction with high MW of *N*. *atra* venom under non-reducing conditions but weak signals were seen under reducing conditions. However, *B*. *multicinctus* antivenin showed strong immunoreactivity with high MW of *O*. *hannah* venom both under non-reducing and reducing conditions. Since no anti-king cobra venom antiserum available in China, using single *N*. *atra* antivenin / *B*. *multicinctus* antivenin or combining both antivenins to treat *O*. *hannah* envenomation was recommended by China Expert Consensus on the management of snake-bites and has practiced in clinic for many decades in China [39]. Thus, present results strongly indicated that high MW of *O*. *hannah* venom might contribute in the lethal activity of the venom. Furthermore, our previously reported L-amino acid oxidase [51] and blood coagulation factor X activator [52] represented the high MW of *O*. *hannah* venom components that could be reacted with commercial *B*. *multicinctus* antivenin were determined. The L-amino acid oxidase and blood coagulation factor X activator are identical to UniProt database of P81383 and A3R0T9 respectively by *de novo* MS/MS sequencing (S5 Fig).

## Conclusions

In accordance with previous results that β-BGTs are the most lethal and abundant components contained in the *B*. *multicinctus* venom. The determined ED_50_ values of prepared β-BGT antiserum and commercial *B*. *multicinctus* antivenin against *B*. *multicinctus* venom demonstrated that prepared β-BGT antiserum showed a better animal protection efficiency over commercial *B*. *multicinctus* antivenin (12 mg/kg vs 17.68 mg/kg). Our present results suggested that it might be feasible for commercial *B*. *multicinctus* antivenin producer to add a single gel filtration step and us both β-BGT and α-BGT fractions to produce the *B*. *multicinctus* antivenin to largely avoid unnecessary high MW reaction antibodies (Figs 4B and S1). The LD_50_ values of purified α-, β- and γ-BGTs via *i*.*p*, *i*.*m* and *i*.*v* injection routines were systematically determined in Kunming mice. Administration routines did have effects on the lethal activity of different bungarotoxins were first revealed. Importantly, the recommended using *B*. *multicinctus* antivenin to treat king cobra victims was supported but using *B*. *multicinctus* antivenin to treat *B*. *fasciatus* envenomation in clinic practice in China was not supported by present evidences.

## Supporting information

S1 Fig Purification of bungarotoxins from *B. multicinctus* venom. The purification process of β-bungarotoxin (A-D). The purification process of α-bungarotoxin (A, E, F). The purification process of γ-bungarotoxin (A, E, G). The buffer used in the experiments were PBS (A), sodium acetate-acetate buffer solution (0.05M, pH 5.0) (B, E), phosphate buffer solution (0.02M, pH 7.3) (C).
(TIF)

S2 Fig MS/MS *de nove* sequencing of the bungarotoxins by MALDI-TOF/TOF. The bungarotoxins were digested by trypsin. The MS/MS data of α-BGT (A). The MS/MS data of β-BGT (B). The MS/MS data of γ-BGT (C). Mass tolerance for MS/MS ions spectrum was±0.5 Da.
(TIF)

S3 Fig SDS-PAGE and Western blot of each fractions in S1B Fig. SDS-PAGE under non-reducing conditions, 1.5 μg/sample (A). Western blot revealed by prepared anti-β-BGT antiserum under non-reducing conditions, 1.5 μg/sample (B).
(TIF)

S4 Fig Expression and purification of MBP-γ-BGT recombinant protein. M: Molecular marker. 1: Whole lysate of uninduced cells. 2: Whole lysate of IPTG induced cells. 3: Periplasmic extract. 4: Purified proteins from periplasmic extracts by amylose affinity column eluted with maltose.
(TIF)

S5 Fig MS/MS *de nove* sequencing of the L-amino acid oxidase and blood coagulation factor X activator from *O*. *hannah* venom by MALDI-TOF/TOF. SDS-PAGE of *O*. *hannah* venom under non-reducing conditions, 25 μg/sample (A). Western-blot profile of commercial *B*. *multicinctus* antivenin against *O*. *hannah* venom (B). The MS/MS data of L-amino acid oxidase and blood coagulation factor X activator (C). Mass tolerance for MS/MS ions spectrum was±0.5 Da.
(TIF)
