# Supplementary figures and images for: Immunoreactivity and neutralization study of Chinese Bungarus multicinctus antivenin and lab-prepared anti-bungarotoxin antisera towards purified bungarotoxins and snake venoms
Source: PLoS Negl Trop Dis. 2020 Nov 30;14(11):e0008873. doi: 10.1371/journal.pntd.0008873 (PMC7728252; doi:10.1371/journal.pntd.0008873)

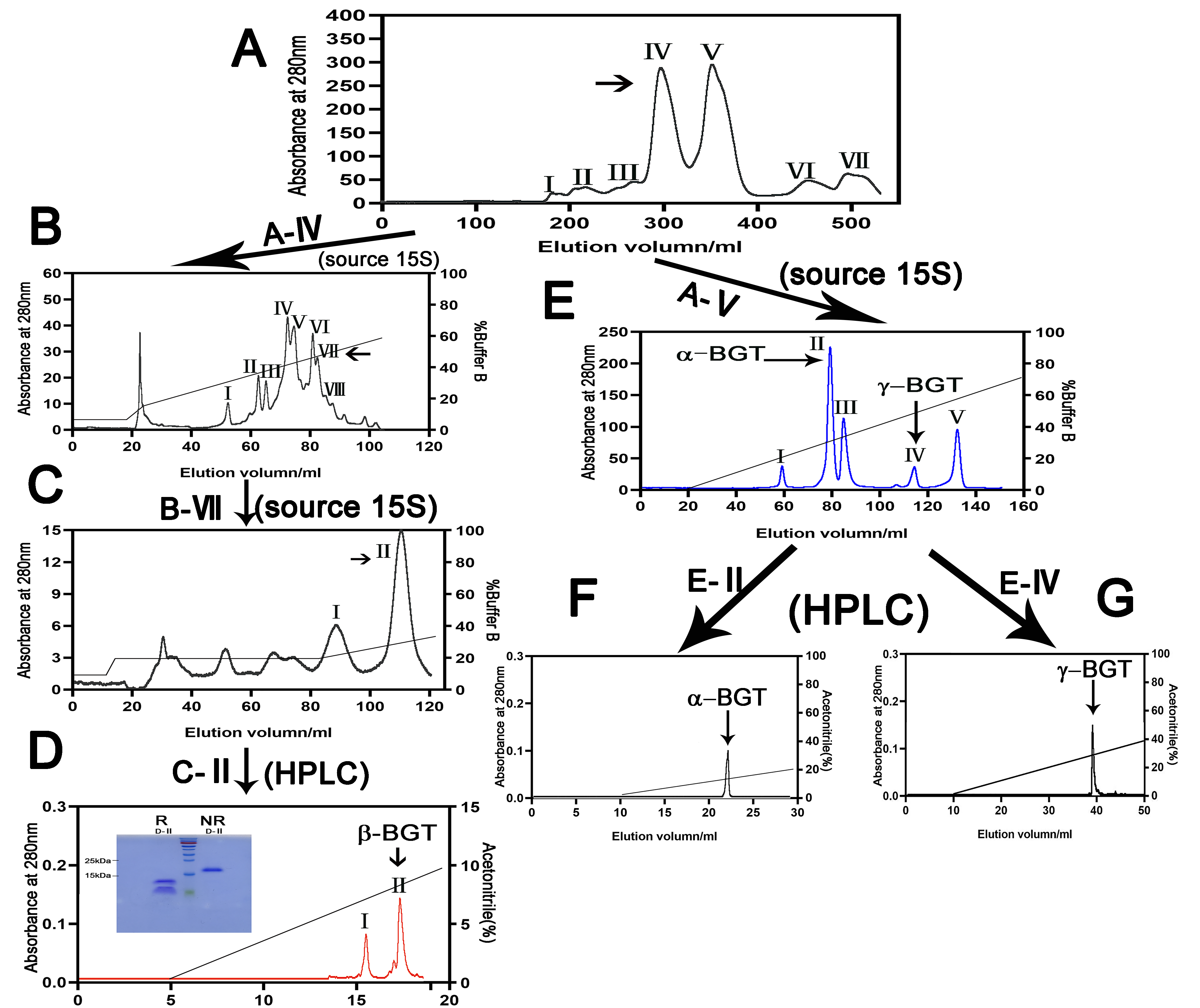

Supplement: S1 Fig — The purification process of β-bungarotoxin (A-D). The purification process of α-bungarotoxin (A, E, F). The purification process of γ-bungarotoxin (A, E, G). The buffer used in the experiments were PBS (A), sodium acetate-acetate buffer solution (0.05M, pH 5.0) (B, E), phosphate buffer solution (0.02M, pH 7.3) (C). (TIF) [file pntd.0008873.s001.tif]

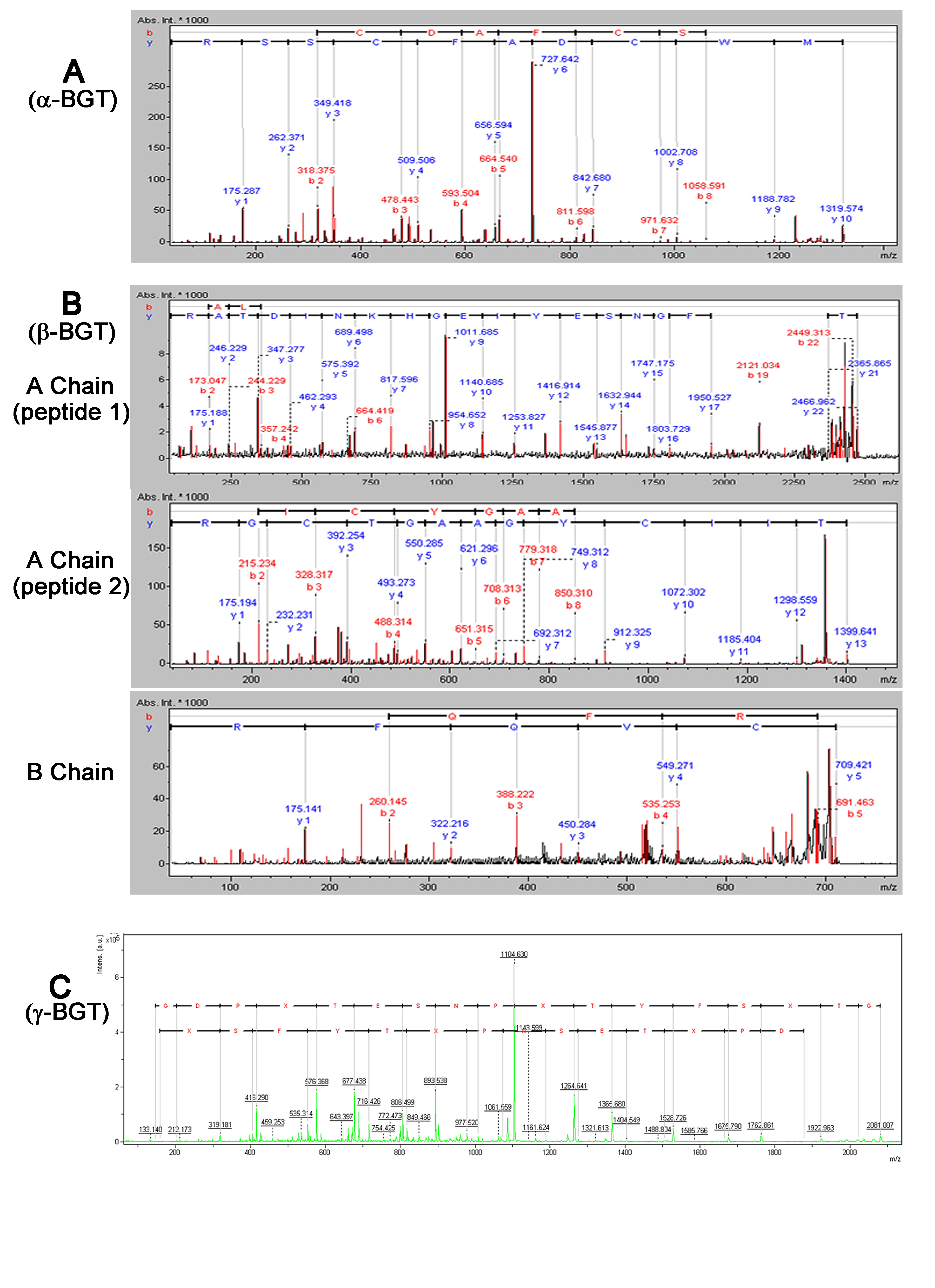

Supplement: S2 Fig — The bungarotoxins were digested by trypsin. The MS/MS data of α-BGT (A). The MS/MS data of β-BGT (B). The MS/MS data of γ-BGT (C). Mass tolerance for MS/MS ions spectrum was±0.5 Da. (TIF) [file pntd.0008873.s002.tif]

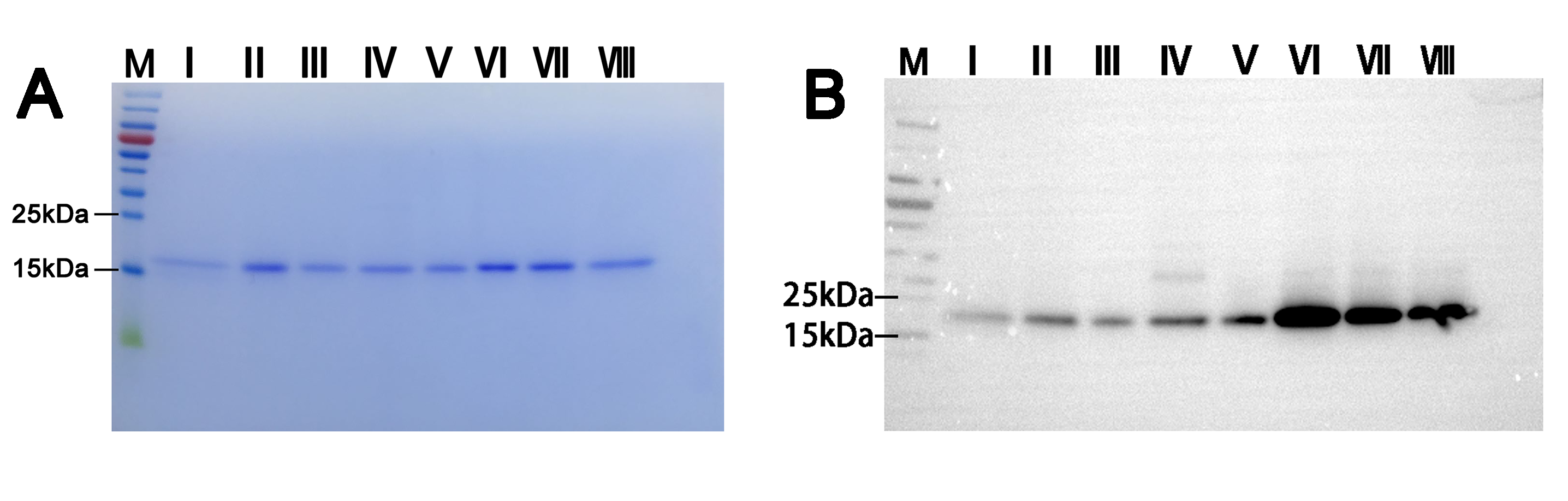

Supplement: S3 Fig — SDS-PAGE under non-reducing conditions, 1.5 μg/sample (A). Western blot revealed by prepared anti-β-BGT antiserum under non-reducing conditions, 1.5 μg/sample (B). (TIF) [file pntd.0008873.s003.tif]

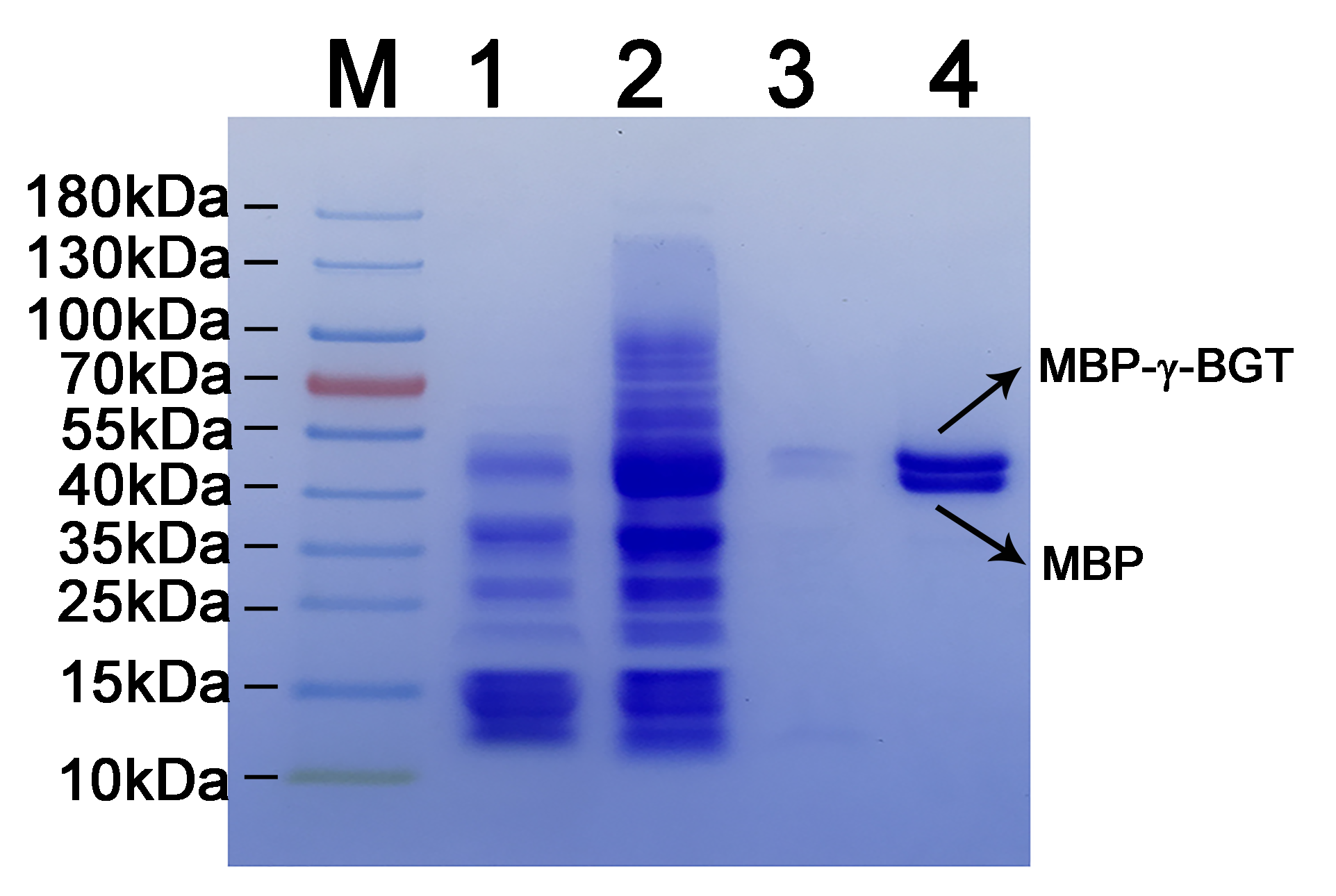

Supplement: S4 Fig — M: Molecular marker. 1: Whole lysate of uninduced cells. 2: Whole lysate of IPTG induced cells. 3: Periplasmic extract. 4: Purified proteins from periplasmic extracts by amylose affinity column eluted with maltose. (TIF) [file pntd.0008873.s004.tif]

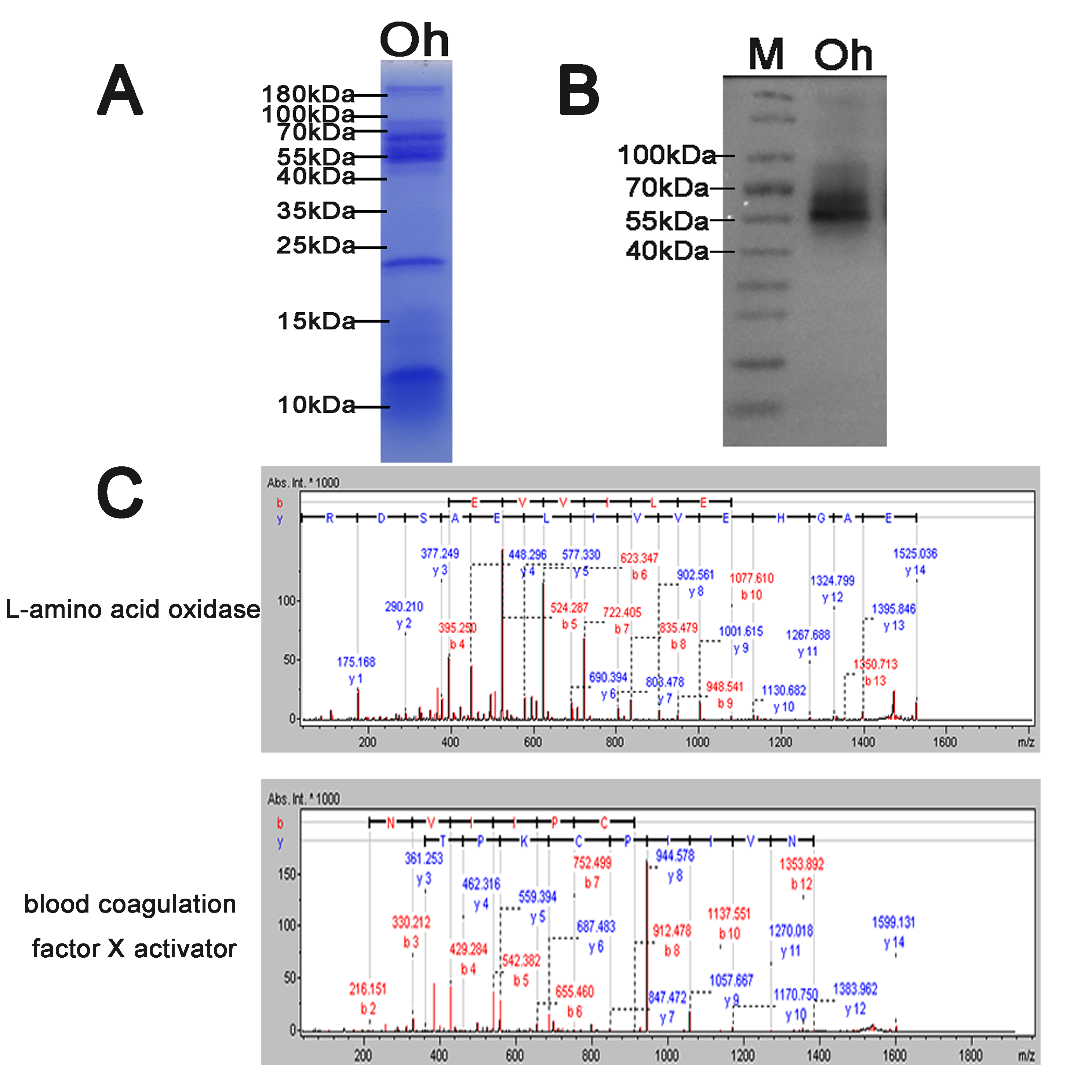

Supplement: S5 Fig — SDS-PAGE of O. hannah venom under non-reducing conditions, 25 μg/sample (A). Western-blot profile of commercial B. multicinctus antivenin against O. hannah venom (B). The MS/MS data of L-amino acid oxidase and blood coagulation factor X activator (C). Mass tolerance for MS/MS ions spectrum was±0.5 Da. (TIF) [file pntd.0008873.s005.tif]
